# Convection-Enhanced Delivery in Malignant Gliomas: A Review of Toxicity and Efficacy

**DOI:** 10.1155/2019/9342796

**Published:** 2019-07-22

**Authors:** Minghan Shi, Léon Sanche

**Affiliations:** ^1^Department of Radiation Oncology, The Second Affiliated Hospital of Zhejiang University, School of Medicine, Hangzhou, China; ^2^Department of Nuclear Medicine and Radiobiology, Université de Sherbrooke, Sherbrooke, QC, Canada

## Abstract

Malignant gliomas are undifferentiated or anaplastic gliomas. They remain incurable with a multitude of modalities, including surgery, radiation, chemotherapy, and alternating electric field therapy. Convection-enhanced delivery (CED) is a local treatment that can bypass the blood-brain barrier and increase the tumor uptake of therapeutic agents, while decreasing exposure to healthy tissues. Considering the multiple choices of drugs with different antitumor mechanisms, the supra-additive effect of concomitant radiation and chemotherapy, CED appears as a promising modality for the treatment of brain tumors. In this review, the CED-related toxicities are summarized and classified into immediate, early, and late side effects based on the time of onset, and local and systemic toxicities based on the location of toxicity. The efficacies of CED of various therapeutic agents including targeted antitumor agents, chemotherapeutic agents, radioisotopes, and immunomodulators are covered. The phase III trial PRECISE compares CED of IL13-PE38QQR, an interleukin-13 conjugated to* Pseudomonas aeruginosa* exotoxin A, to Gliadel® Wafer, a polymer loaded with carmustine. However, in this case, CED had no significant median survival improvement (11.3 months vs. 10 months) in patients with recurrent glioblastomas. In phase II studies, CED of recombinant poliovirus (PVSRIPO) had an overall survival of 21% vs. 14% for the control group at 24 months, and 21% vs. 4% at 36 months. CED of Tf-diphtheria toxin had a response rate of 35% in recurrent malignant gliomas patients. On the other hand, the TGF-*β*2 inhibitor Trabedersen, HSV-1-tk ganciclovir, and radioisotope ^131^I-chTNT-1/B mAb had a limited response rate. With this treatment, patients who received CED of the chemotherapeutic agent paclitaxel and immunomodulator, oligodeoxynucleotides containing CpG motifs (CpG-ODN), experienced intolerable toxicity. Toward the end of this article, an ideal CED treatment procedure is proposed and the methods for quality assurance of the CED procedure are discussed.

## 1. Introduction

Despite the fast development of several modalities for cancer treatment, such as chemotherapy, immunotherapy, and targeted therapy, pharmaceutical agents available for brain tumor treatment remain rare. The failure of the application of these agents in brain tumor is partially due to the existence of the blood-brain barrier (BBB), which prevents them from entering the tumor site. This problem led to the development of strategies to open temporarily the BBB, including osmotic and ultrasonic BBB disruption [1–4]. During the osmotic BBB disruption procedure, hyperosmotic agents such as mannitol are infused and a temporary cell membrane retraction is induced, creating a physical opening between the endothelium cells [1–3]. In the case of ultrasonic BBB disruption, pulsed ultrasound is applied in combination with infusion of microbubbles to disrupt the BBB, thus increasing the intratumoral concentration of the therapeutic agents [4]. Rather than disrupting the BBB, another strategy consists of bypassing the BBB. In the early 1990s, Oldfield and his research team proposed a new technique to traverse the BBB, convection-enhanced delivery (CED) [5], by which interstitial infusion of the agent by a syringe pump creates a pressure gradient, permitting enhanced distribution of the brain. The technical parameters of the CED procedure have been reviewed by Allard E. et al. [6].

With the emergence of novel radiation therapy techniques, such as intensity-modulated radiation therapy (IMRT), volumetric-modulated arc therapy (VMAT), and 4*π* radiation therapy (RT) [7], the precision of target volume delineation has largely improved, which in some aspects could make CED of therapeutic agents less attractive. However, the rationale behind CED remains a potentially useful way to enhance drug delivery to the brain. Besides increased tumor uptake of antitumor agents, by penetration of the BBB [8], the localization of the drug provides an effective condition for concomitant chemoradiation therapy and the benefits of its supra-additive effect [8, 9].

## 2. Preclinical Studies

In animal studies, two delivery systems were commonly employed for safety and efficiency evaluation. (1) A micro infusion syringe connected to a micro infusion pump: Our group optimized this technique by using flat tip 33 Ga needle gas-tight Hamilton syringe, to inject 10 *μ*L of therapeutic agent at an infusion rate of 0.5 *μ*l/min, for a duration of 20 mins [8]. These parameters prevented reflux from the injection site, permitting a large distribution volume in the tumor site of a rat brain. (2) An ALZET® osmotic pump, a device embedded subcutaneously: It provides an infusion time of up to a week [10, 11] and hence has the advantage of long-term infusion without recurrent operations. The longer infusion time means less reflux and a larger diffusion volume, which is a key parameter for brain tumor CED in humans. Since the brain volume of a rat at 8 weeks is only ~600 mm^3^ [12], the difference in the distribution volume, after 20 mins infusion, with a syringe or an osmotic pump can be indistinguishable. Yang et al. compared survival after CED injection of carboplatin in F98 glioma bearing rats with a syringe, to that obtained with an ALZET® osmotic pump. The median survival time (MeST) for controls was 23 days after cell implantation. CED of carboplatin with the syringe extended the MeST to 46 days, whereas the ALZET® osmotic pump further increased it to 59 days. However, the osmotic pump delivered 84 *μ*g of carboplatin, as opposed to 20 *μ*g by the CED with the syringe [13], suggesting that prolonged administration is therapeutically more effective.

In experimental studies, catheter design plays an important role in reducing the reflux of the infusate and increasing the convection volume [14]. The most common is the one port catheter, which consists of a cannula with one port at the tip. This catheter has been well studied in gels and is widely used in animal studies [15–17]. The computational and experimental studies in gels and rats showed that reflux decreases as the diameter of the needle decreases [15, 18, 19]. Thus, to prevent reflux within the catheter, a diameter smaller than 30 Ga was usually chosen, which limited the flow rate to 0.5 *μ*L/min [20, 21]. To further reduce reflux, Krauze and his colleagues designed a stepped cannula [22]. Compared to a simple 32 gauge needle, the step-design cannula was able to increase the reflux-free flow rate from 5 *μ*L/min to 50 *μ*L/min in agarose gel and from 0.5 *μ*L/min to <50 *μ*L/min in the rat brain [15, 17, 19]. To increase the distribution volume, multiple-pore catheter was designed. It has five pores of 0.2 mm diameter on opposite sides of the tip [23]. Computational analysis predicted that the drug distribution from the multiple pores design in the caudate nucleus increased the distribution volume by 26%. However, an experimental study with this type of catheter in gels has shown that the infusate can only be released from the proximal pores leaving the rest of the pores useless [24]. Another design increased the distribution volume by using a hollow fiber catheter, with millions of nanoscale pores (450 nm) [25]. Seunguk and his colleagues found that the distribution volume of a dye injected in a gel by such a hollow fiber catheter was 2.7 times larger than that obtained with a one port catheter. However, further studies are required for clinical applications, because longer catheters would be required in humans, and the physical characteristics of the drug distribution may change. In clinical settings, before the administration of chemotherapy, a cavity is left by tumor resection, which makes the administration of a chemotherapeutic agent by CED complicated. To alleviate the problem, a balloon-tipped catheter with an inflatable balloon attached to the tip of the catheter was designed. The inflatable balloon fills the resection cavity and thus reduces reflux [26, 27].

Halle et al. systemically reviewed preclinical CED studies and found that methodological parameters such as catheter design, infusion rate, and infusion duration varied among different studies. Data on endpoint measurements of drug diffusion and adverse effects are often missing in many preclinical studies [28]. These parameters are crucial for carrying out preclinical investigations and further clinical studies based on promising results from animal experiments. The authors also suggested that* in vivo* studies with larger animal brains should be carried out before undertaking clinical trials.

Besides the different techniques employed in CED, different types of medications have been tested: chemotherapeutic drugs, antibodies, toxins, vaccines, etc. The standard treatment of glioblastomas (GBM), the most common and aggressive glioma in adults, is composed of several combined modalities, which may include surgery, RT, concomitant and adjuvant temozolomide (TMZ) chemotherapy, and more recently alternating electric field therapy. The current standard treatment includes concurrent and adjuvant TMZ chemotherapy and it is therefore reasonable to compare CED with this agent in preclinical studies. Saito et al. in 2004 studied CED delivery of tumor necrosis factor-related apoptosis-inducing ligand (TRAIL) and systemic delivery of TMZ in a U87MG intracranial xenograft rat model [29]. Both CED of TRAIL and systemic delivery of TMZ increased survival. More importantly, the synergistic effect of the chemotherapeutic agent cisplatin and TRAIL has been demonstrated in a glioma model. In this study, a combination of CED of TRAIL with systemic delivery of TMZ further increased the survival (P = 0.032) [30]. Barth and his research team in 2011 studied RT plus oral delivery of TMZ in daily doses of 80 mg/ kg body weight for 5 d or CED of 1.5 mg TMZ in 15 *μ*L at a flow rate of 0.5 *μ*L/min for 30 min in F98 glioma bearing Fischer rats [13]. Radiation was performed at 6, 7, and 8 days after implantation with a daily dose of 5 Gy. They obtained an MeST of 23 days for oral TMZ plus radiation and 27 days for CED of TMZ plus radiation, compared to 21 days for radiation alone. Although a modest increase of MeST in the CED group was observed, no significant difference was found. Indeed, due to its inherent ability for crossing the BBB, TMZ may not be a good candidate for CED and the study of Barth and his research group confirmed this hypothesis. Other studies focused on a nanocarrier for the delivery of TMZ by CED, i.e., the polymeric nanoparticle vector [31], TMZ-loaded photopolymerizable PEG-DMA-based hydrogel [32], and liposomes [33]. They all demonstrated various degrees of antitumor efficacy compared to free TMZ or reduced toxicity to normal brain, but failed to demonstrate the advantage of CED of TMZ over oral delivery of TMZ, which is the standard method of administration in the clinic.

The well-studied chemotherapeutic agents by CED are platinum-based drugs, such as cisplatin, carboplatin, and oxaliplatin, delivered in glioma bearing rats which were largely investigated by the groups of Barth and Elleaume [11, 13, 20, 21, 34–36], Lonser [17], Tomita [37], and ours [8, 38, 39]. These authors measured the combined effect of radiation and platinum drugs. However, due to differences in tumor model, doses of infusion, and protocol design, the effectiveness of these drugs cannot be determined from a comparison of the results of the different groups. Moreover, CED of these drugs was not compared to other routes of delivery such as intra-arterial injection and intravenous infusion. For this reason, in our laboratory, we performed a series of studies comparing intravenous (iv), intra-arterial (ia), and CED of different platinum drugs, as well as their combinational effect with radiation [8, 39–41]. It was further observed that carboplatin was the most effective platinum drug compared to cisplatin and oxaliplatin. When encapsulated within a liposome, carboplatin still had the advantage over the others; however, the other platinum drugs were not encapsulated nor designed for CED.

There are four main theories regarding the mechanisms of the synergistic action of platinum drugs and radiation: radiation sensitization of the hypoxic cells by platinum drugs [42–45]; fixing by platinum drugs of the radiation-induced sublethal DNA damage [46, 47]; radiation-induced formation of toxic platinum adducts [48, 49]; and direct radiation sensitization by platinum drugs [50–54].

Tippayamontri et al. studied the amount of DNA-platinum adducts formed in the nucleus of cancer cells over time, both in vitro and in vivo. The efficiency of RT was found to be proportional to the amount of the Pt drug bound to the DNA of the cancer cells. When mice bearing a human colorectal HCT116 tumor were irradiated at the time of highest yields of DNA-platinum adducts, the synergy between radiation and cisplatin or oxaliplatin and their liposomal formulation was the largest [55, 56]. Based on their findings, CED of these agents was carried out to further increase tumor uptake. However, survival increase of F98 glioma bearing Fisher rats was not significant. This may reflect the ceiling of radiation enhancement based on DNA-platinum yield [8].

## 3. Clinical Studies

### 3.1. Clinical Protocols

Before carrying out clinical trials with CED, a carefully designed clinical protocol is surely a prerequisite to achieve significant results, especially for new treatment techniques, where many parameters need to be adjusted from animal to human protocols. The catheter designs and placement, flow rate, choice of therapeutic agent, infusion volume, and visualization of the infusion volume are all key parameters that need to be accurately assessed. Considering the problems encountered in the setting of these parameters, but not limited to them, the PRECISE phase III clinical trial failed to demonstrate an advantage of CED over standard-of-care treatment [57].

Ren et al. in 2003 designed and published a phase I/II protocol of CED of a liposomally encapsulated replication-disabled Semliki Forest virus vector, carrying the human interleukin 12 gene (LSFV-IL12). This protocol involved treatment of recurrent or progressive GBM to evaluate the safety, maximum tolerated dose (MTD), and antitumor efficacy [58]. They designed an infusion volume of 11 mL at a maximum infusion rate of 0.5 mL/h for a total of 24 h. However, the results of this study were not further analyzed and disseminated.

Another phase I clinical trial protocol was proposed by White et al. in 2012, after a series of successful animal studies of CED of carboplatin [59]. The principal research objectives were to determine the safety, tolerability, and MTD, via dose escalation and further facilitate the safe application of a phase II protocol. In addition, the efficacy, carboplatin distribution, and visualization of infusate will also be evaluated with a carboplatin delivery of 8 h/d for 3 consecutive days at a maximum infusion rate of 0.6 mL/h for no more than 20 mL of infusate per day.

### 3.2. Toxicity Studies in Clinical Trials

The safety and tolerability of various therapeutic agents, including antibodies, targeted toxins, interleukins, chemotherapeutic drugs, targeted radioisotopes, and vaccines (Table 1), have been studied in clinical trials in the last two decades.

Based on their results of intratumoral injection of monoclonal antibodies in patients with advanced malignant glioma, Wersall et al. classified side effects as immediate (<2 h) or late (5-48 h) and we believe that CED could have a similar evolution [60]. On the other hand, Kunwar S. et al. defined three phases of toxicity based on the time of onset: pre-CED, peri-CED, and post-CED [61]. Here, based on their classifications and the review of all published CED clinical trials, we reclassify CED-related toxicities as immediate, early, and late side effects.

(a) Immediate side effects occur within hours of the placement of catheters. Physical damage to the brain tissue and cerebral hemorrhage by the catheter are possible causes related to symptoms such as headache, seizure, and neurological toxicities [61].

(b) Early side effects occur hours to days after CED. Mechanical stress caused by the infusion of fluid leads to common complaints such as headache, seizure, worsening of neurological symptoms, shivering, and mild fever [62, 63].

(c) Late side effects include mainly neurological toxicity, due to the toxicity from delivered drugs, occurring days to weeks after infusion [64–67].

Depending on the location of the toxicities related to CED, we summarized them into two categories. 

*(a) Local Toxicities (Common and Severe)*. These comprise neurological toxicities due to inflammatory reactions, necrosis, and peritumor edema [64]. Depending on the location of the tumor and site of infusion, patients could manifest different types of neurological toxicity symptoms: headache, seizure, nausea, pyrexia, sensory disturbance, upper motor neuron lesion, aphasia/speech disorder, and memory impairment [68]. The reaction can be severe and cannot be satisfactorily controlled by steroids, and debulking is needed to reduce the mass effect [65]. In the study by Rand et al., nine patients received 30-185 mL of IL-4(38-37)-PE38KDEL and seven of them required craniotomy due to uncontrollable cerebral edema. The reaction seems not related to the infusion rate, infusion volume, total infused dose, and number of catheters. The edema appeared 10-97 days after CED procedure; thus, it was not procedure-related. However, edema could be well controlled by steroids [64, 69] in other trials. In the procedure of Weaver and Laske, 5-180 mL of Tf-CRM107, a targeting toxin, was infused in patients with anaplastic oligodendroglioma (AO)/anaplastic astroglioma (AA)/GBM. The symptoms related to the edema and mass effect were fewer (i.e., 3 in 44 treatments) and well controlled by steroids and hyperosmolar therapy. Their phase II clinical trial demonstrated a similar toxicity and showed that cerebral edema can be well controlled by medical treatment [70]. With a similar pretreatment and treatment conditions, such large differences in the rate and severity of local toxicity can only be explained by the infused agent.

Local infection is related to the placement of the catheter and infusion time. Klatzmann et al. identified the pathogens to be gram negative and staphylococcus bacteria, due to the catheter and CSF leak to the skin. These infections were controllable with antibiotics [67, 69, 71–75]. Complications such as subdural empyema and bacterial meningitis were diagnosed in the study by Lidar et al. [67]. Chemical meningitis happens when chemotherapeutic agents, such as Taxol, reflux from the infusion site (i.e., 40% of patients experienced chemical meningitis) [67]. 

*(b) Systemic Toxicities (Rare and Transitory)*. Studies with TP-38 or IL13, IL4, and Trabedersen did not show any systemic toxicity. General toxicities expressed as fever, fatigue, and erythema were observed [74, 76, 77], as well as gastrointestinal symptoms (nausea, vomiting) [76–78]. Hematological changes (decreased WBC, platelet, lymphopenia [63, 77, 78]) and liver enzyme perturbations (elevated AST, ALT, LDH, CRP, hypoalbuminemia [63, 64, 74, 77]) were also observed.

### 3.3. Efficacy Studies in Phase II and Phase III Clinical Trials

The efficacy of CED clinical studies in GBM treatments was reviewed by Jahangiri in 2017 [79]. Our section includes, in addition to all clinical trials reviewed by this author, more recent ones published after 2017 and those related to other malignant gliomas.

#### 3.3.1. Targeted Antitumor Agents

*Tf-CRM107 (TransMID) (Tf-Diphtheria Toxin) [64, 70]*. This agent is a human transferrin, which targets receptors on the surface of tumor cells fused to a diphtheria toxin. In the phase I/II study of Laske group, out of 15 evaluated recurrent malignant gliomas patients, seven had partial response (PR) and two even had a complete response (CR) to Tf-CRM107 with a dose of 0.5-199 *μ*g per treatment at a maximum infusion rate of 0.24-0.6 mL/h, giving a total volume of 5-180 mL. The tumor response appeared to be dose-dependent, with two out of five of the evaluated patients having PR at the dose of 0.5-12.8 *μ*g, while in the higher dose groups, seven patients out of 10 had PR or even CR. Thus, the same group carried out the phase II study at a total dose of 26.8 *μ*g per treatment, at an infusion rate of 0.4 mL/h, for a total of 40 mL. In the 34 evaluated patients, five patients had CR, seven had PR, which was a 35% response rate. However, all patients enrolled had a progressive disease and the progression of 9 of them was halted due to the response to treatment. Moreover, the magnetic resonance imaging (MRI) response rate was correlated with the survival analysis, with a median survival of 37 weeks. Due to these encouraging results, a phase III clinical trial of recurrent GBM was planned.

*IL13- PE38QQR [57]*. This agent is a human interleukin-13 (IL13) conjugated to a modified form of* Pseudomonas aeruginosa* exotoxin A (PE38QQR). The tolerable toxicity profile and efficacy over control groups, demonstrated by a series of phase I studies, led to the design of a phase III trial, also known as the PRECISE trial [61, 68, 80, 81]. It compared survival of CED of IL13-PE38QQR with tumor cavity placement of Gliadel® Wafer (GW). There were 296 recurrent GBM patients recruited; 192 were assigned to the CED group and 104 to the GW group. Infusion was performed at rate of 0.75 mL/h for 96 hours with a concentration of 0.5 *μ*g/mL, which is the MTD assigned from safety studies. Unfortunately, of the patients evaluable for efficacy, the median survival for the CED group was 11.3 months compared to a median survival of 10 months in the GW group. No statistical significance was found (P = 0.310; hazard ratio 0.81; 95% CI = 0.67-1.18).

The underlying reason of the failure of this multicenter study was further analyzed [82]. Catheter positioning data were retrieved and the distribution volume of the infusate was predicted through iPlan® Flow software from Brainlab. The prediction showed that only 20.1% of peritumoral area was covered by IL13- PE38QQR. However, the effect size of the catheter score and the number of optimally positioned catheters on PFS are small. Thus, before carrying out further clinical protocols, the technical problems must be solved and quality control must be first assured, especially the optimization of parameters, such as geometry of the infusion catheter, flexibility of protocol, and determination of drug distribution. 

*HSV-1-tk GCV [71, 83–85]*. This regimen was a two-step treatment modality. Herpes simplex virus thymidine kinase (HSV-tk) gene was first transduced to the glioblastoma cells by either intratumoral injection or CED, then ganciclovir (GCV) was delivered systemically. Several investigations with intracerebral infusion of HSV-1-tk failed to demonstrate survival benefits due to limited diffusion volume of HSV-1-tk. Later in 2003, Voges et al. designed a liposome encapsulated HSV-1-tk and delivered it through CED with the expectation of an augmented distribution volume [62]. Unfortunately, it was not the case: their large and positively charged liposomes remained at the site of infusion, as observed in our recent study [39]. As a result, only two patients out of eight had PR. 

*TGF-β2 Inhibitor Trabedersen (AP 12009) [75, 86]*. This compound is a transforming growth factor 2 (TGF-*β*2) inhibitor. It was evaluated in 145 recurrent/refractory AA/GBM patients by Bogdahn et al. Patients were assigned to 2 different dose groups, 2.48 mg for 10 *μ*M group and 19.81 mg for 80 *μ*M group at an infusion rate of 0.24 mL/h for 7 days [75]. In the subgroup analysis, compared to conventional chemotherapy (TMZ or procarbazine/CCNU/vincristine (PCV)), Trabedersen increased the MeST of the AA subgroup from 21.7 months with conventional chemotherapy to 39.1 months (but not significant); similar MeST CED and conventional chemotherapy were also obtained from the GBM subgroup. 

*Recombinant Poliovirus (PVSRIPO) [87]*. This is an attenuated poliovirus type 1 vaccine. Poliovirus receptor CD115 is expressed in glioblastoma and the recombinant poliovirus can recognize CD115 and infect the tumor cells creating a cytotoxic effect. Sixty-one recurrent GBM patients were recruited in this phase II trial, during which, PVSRIPO was infused at a rate of 0.5mL/h for 6.5 hours. The results were compared to a historical control group of 104 patients. Although no significant improvement of the median survival time was observed for CED of PVSRIPO (12.5 months) compared to the control group (11.3 months), the survival of the PVSRIPO group reached a plateau starting at 24 months. The overall survival at 24 months is 21% for CED of PVSRIPO vs. 14% for control group, and at 36 months it is 21% vs. 4%. Moreover, two of them had survived over 69 months after infusion. Thus, further investigations on this vaccine are warranted.

#### 3.3.2. Chemotherapeutic Agent

*Paclitaxel [67, 69, 72]*. This agent is a conventional chemotherapeutic drug that interferes with microtubules during cell division. Its antitumor effects on brain tumors have been reported in preclinical studies. Since it does not pass the BBB, it was considered as an interesting compound for treatment via CED. In the study by Lidar et al., 11 out of 15 patients had either PR or CR. The distribution volume after CED was confirmed by diffusion-weighted MRI (DW-MRI). They infused paclitaxel 3.6-7.2 mg/day for 5 days at an infusion rate of 0.3 mL/h. However, 40% of patients had chemical meningitis and 20% had neurological deterioration. Another group performed a similar study but followed with 18F-fluoro-ethyl-tyrosine-positron emission tomography (18F-FET-PET) [69, 72]. They employed similar parameters as Lidar et al., i.e., a total dose of 18 mg (0.5 mg/mL) or 9 mg (0.25 mg/mL) at an infusion rate of 0.3 mL/h for 120 hours. Six out of eight patients had a temporally stable disease. They also demonstrated that FET-EPT is better than MRI to follow-up patients and that an increase in FET uptake almost always implies a recurrence of the tumor.

#### 3.3.3. Radioisotopes

^131^ *I-chTNT-1/B mAb (Cotara) [76]*. This compound is a ^131^I-labeled chimeric monoclonal antibody that targets the intracellular antigen histone H1, which is exposed in the necrotic core of gliomas. In a phase II clinical trial, 39 patients were recruited, but only 12 recurrent glioblastoma patients who had received a dose in the therapeutic range (1.25-2.5 mCi/m^3^) were evaluated. Among the 12 patients, only one patient had PR and 6 patients had stable disease. Necrotic tumors, which can be pathologically distinguished through features such as fibrinoid necrosis of blood vessel wall, white matter necrosis, and telangiectasis [88], were seen in several reoperated patients, which proved that Cotara has radiation effect on tumor, but its efficacy needs further evaluation.

#### 3.3.4. Immunomodulators

*Oligodeoxynucleotides Containing CpG Motifs (CpG-ODN) [74]*. CpG-ODN is a strong immunomodulator, which activates both innate immunity (natural killing cells and macrophages) and adaptive immunity with the expectation of targeting tumor cells by the immune system. In the studies carried out by Carpentier et al. [63], CpG-ODN was infused at a rate of 0.2 mL/h for 5 hours. The phase I study demonstrated a tolerated dose of 20 mg [63]. In the following phase II trials, there was only one partial responder and three minor responders in 31 patients. Even worse, 13 patients out of 31 had treatment related seizure, among whom 3 had generalized seizure.

Overall, few efficacy studies demonstrated superior survivals over standard treatment regimen. Future investigations should adjust and standardize methodological parameters. Distribution volume prediction and validation should be implanted. Besides the above-mentioned reasons, another possible contributing factor for the failure of the clinical trials is related to the fact that CED augments the interstitial pressure, which could enhance the invasion of glioma cells [89].

## 4. Discussion

### 4.1. Choice of Agent

As we mentioned above, the advantages of CED compared with modern RT depend on the type of agents, i.e., those with different functions, such as targeted toxins, radiosensitizing chemotherapeutic agents, radioisotopes, and so on. CED of these agents aims to gain local control of tumor progression, as does RT, with a different approach, which includes tumor cell targeting and radiosensitization. A choice of a suitable agent can add valuable antitumor efficacy to traditional RT. Such agents should have the ability to selectively target the tumor cells in infiltrative areas and thus spare normal brain cells. In this case, higher local doses can be obtained in cancerous tissue compared to conventional RT, where toxicity of normal tissue is a major concern. In fact, the recurrent tumor usually emerges from the peritumoral area. When compared to recently developed immunotherapeutic methods, CED offers a novel approach for the application of those therapeutic agents to malignant gliomas [94, 95].

### 4.2. Treatment Planning

Under ideal conditions, the medical team planning clinical studies employing CED should be similar to that in charge of radiation treatments. In the latter, radiation oncologists delineate the gross tumor volume (GTV) based on CT scans and/or MRI, clinical targeting volume (CTV) 1 and 2 for high and low risk area and organs, respectively. They assign the treatment dose to each target volume restricting, as much as possible, the dose to organs at risk. Medical physicists or dosimetrists then calculate and design a treatment plan to meet the requirements of radiation oncologists. The dose planning is based on the angle and number of the radiation fields, CT value of the tissue, and so on. While in the future treatment planning for CED will first be based on MRI, many other parameters will have to be considered. These should include GTV, peritumoral area, organs at risk, such as brain stem, hippocampus, and other areas related to functions that influence the quality of life. Treatment would benefit from software, such as iPlan® Flow, based on the DW-MRI, probably with appropriate input parameters such as placement of catheters, surface charge and quantity of drug, viscosity of the infusate, and infusion rate and volume, to simulate the dose assigned for each CTV [96].

### 4.3. Monitoring the Distribution of the Infusate

After treatment planning and simulation, monitoring and validation of the placement of catheters and distribution of infusate are equally important. This procedure is again similar to RT; before each delivery we use cone beam computerized tomography (CBCT) system to ensure that the targeted tumor is positioned in the planned coordinates. For this purpose, many techniques have been investigated. Coinfusion of gadolinium with therapeutic agents is an easy approach to monitor the infusate distribution, assuming that gadolinium diffuses in the tumor in the same manner as the infusate [97–99]. Loading of gadolinium and drugs in the same vector, such as a liposome, represents another approach to monitoring the distribution [100, 101]. This approach has the advantage of revealing the “true image” of the drug distribution, as long as gadolinium does not leak due to the convection pressure. Methods that do not utilize additional gadolinium appear more attractive, but they may compromise the drug efficacy. Multivoxel ^1^H-MR Spectroscopy through analysis of metabolites ratio of Cho/Cr and Cho/NAA is able to describe the tumor site with and without CED infusion [102], but the resolution of this technique needs to be increased for adequate analysis in the future. DW-MRI is another noninvasive approach that can monitor the response of CED delivery of Taxol [103]. The response can be detected within 24-48 hours with DW-MRI, which is 1-2 days earlier than conventional imaging methods. However, none of the CED clinical trials assessed the distribution efficacy, which may be one of the factors that caused the failure of phase III clinical trial.

## 5. Conclusion

Regardless of emergence of novel therapeutic agents, their application in malignant gliomas remains rare, possibly due to the existence of the BBB. CED bypasses the BBB, increases the tumor uptake, and reduces the systemic toxicity. It has made progress during the past 25 years, since its invention, up to phase III clinical trial. It is a clinically feasible procedure with mostly local and tolerable toxicity, although grade III and IV adverse effects have been reported. Phase II clinical trials of PVSRIPO, Tf-diphtheria toxin, hold promise for future CED studies. Nevertheless, the phase III clinical trial failed to demonstrate survival improvements in the treatment of brain tumors. Analysis of the failure of these clinical trials showed the importance of catheter placement and distribution volume prediction and validation in performing CED treatments. Thus, it appears imperative to carefully analyze the methodological parameters to predict and validate the distribution volume for future clinical studies to be successful.

## Figures and Tables

**Table 1:** Summary of clinical studies of CED in the treatment of malignant gliomas.

Reference	Therapeutic agent	Trial phase	Diagnosis (n)	Infusion volume	Infusion rate	Treatment responders (n/total pts)	MeST	Drug-related adverse events (rate)
Laske et al., 1997 [64]	Tf-CRM107 (TransMID)	I/II	GBM (9) AA (5) AO (1)	5-180 mL	0.24-0.6 mL/h	9/15	74 wks (responders) 36 wks (non-responders)	Seizures (27%), local toxicity (20%), transit elevation of ALT, AST (93%), mild hypoalbuminemia (80%)

Weaver et al., 2003 [70]	Tf-CRM107 (TransMID)	II	Recurrent AA/GBM (44)	5-180 mL	0.2 mL/h/catheter 2 catheters	12/34	37 wks	Symptomatic progressive cerebral edema (24%), seizure (9%)

Rand et al., 2000 [65]	IL-4 PE38KDEL (NBI-3001)	I	GBM (9)	30-185 mL	0.3-0.6 mL/h	-	-	Headache (11%), seizures (22%), anomia (11%), dysphasia (11%), communicating hydrocephalus (22%), weakness (22%), nausea (11%)

Weber et al., 2001 [66] Weber et al., 2003 [90]	IL-4 PE38KDEL (NBI-3001)	I	GBM (25) AA (6)	40-100 mL		-	-	Headache (45%), seizures (84%), weakness (32%), aphasia (23%), speech disorder (10%), hypoesthesia (16%), coma (10%), wound infection (10%), pyrexia (10%), nausea (23%)

Kunwar et al., 2003 [68] Kunwar et al., 2006 [61] Kunwar et al., 2007 [80]	IL13- PE38QQR	I	MG (5) GBM (46)	19.2-51.8 mL 72 mL	0.4-0.54 mL/h intratumoral 0.75 mL/h intraparenchymal	-	-	Headache (41 %), convulsion (14 %), sensory disturbance (25 %), aphasia/speech disorder (18 %), asthenia (16 %), hemiparesis (14 %), facial paresis (12 %), memory impairment (8 %), pyrexia (8 %), nausea (8 %)

Vogelbaum et al., 2007 [81]	IL13- PE38QQR	I	GBM (22) anaplastic mixed OA (1)	72 mL	0.75 mL/h	-	-	Headache (50%), fatigue (73%), nausea (41%), convulsion (14%), confusion (14%), aphasia (14%), dyspepsia (14%), pyrexia (14%)

Kunwar et al., 2010 [57]	IL13- PE38QQR	III	Recurrent GBM (296)	72 mL	0.75 mL/h	Not reported	36.4 wks IL13- PE38QQR vs. 35.5 wks Gliadel wafer	Headache (0.4%), aphasia (1.2%), hemiparesis (0.8 %), Monoparesis (0.5%), hemiplegia (0.3%), gait disturbance (0.3%), coordination abnormal (0.3%), mental status changes (0.3%)

Sampson et al., 2003 [91] Sampson et al., 2008 [92]	TP-38	I	GBM (17), AO (1), GSC (1), Metastasis (1)	40 mL	0.4 mL/h	-	-	Headache (47%), hemiparesis (20%), speech (20%), constitutional (20%), ocular/visual (13%), seizure (8%)

Voges et al., 2003 [62]	LIPO-HSV-1-*tk* GCV	I/II	GBM (8)	3.5 mL	0.025-0.6 mL/h	2/8	28.1 ± 3.0 wks	Transient worsening of motor aphasia (25%), fever (25%), leukocytosis (25%), ALT (37.5%), AST (25%), LDH (12.5%), CRP (25%)

Lidar et al., 2004 [67]	Paclitaxel	I/II	GBM (13), AA/AO (1), Mixed AO (1)	6-6.6 mL	0.3 mL/h	11/15	32.1 wks	Chemical meningitis (40%), neurological deterioration due to peritumoral edema and necrosis (20%)

Pöpperl et al., 2005 [69] Tanner et al., 2007 [72]	Paclitaxel	II	Recurrent GBM (8)	36 mL	0.3 mL/h	0/8	42.9 wks	Temporary worsening of (pre-existing) neurological symptoms (63%), poor wound healing (25%), neurological deterioration (25%)

Patel et al., 2005 [76]	^131^I-chTNT-1/B mAb (Cotara)	I/II	Recurrent GBM (37) primary GBM (8) AA (6)	4.5-18 mL	0.18-0.72 mL/h	1/12	37.9 wks	headache (14%), convulsions (6%), simple partial seizures (4%), aphasia (6%), weakness (6%), hemiparesis (14%), facial palsy (4%), short-term memory loss (2%), fatigue (6%), nausea (4%)

Sampson et al., 2006 [93]	I^131^-Ch81C6		Recurrent GBM (10)	4.5-18 mL	0.18 mL/h	3/10	30.3 wks	

Pandit-Taskar et al., 2018 [77]	I^124^-8H9	I	Diffuse intrinsic pontine glioma	0.25-4 mL	0.05-0.45 mL/h	-	-	Headache (50%), ataxia (29%), facial palsy (36%), diplopia (25%), muscle weakness (22%), dysarthria (15%), anaemia (32%), platelet count decreased (25%), white blood cells decreased (67%), ALT (32%), AST (25%), hypoalbuminaemia (61%), rash (11%), skin infection (8%), vomiting (18%)

Boiardi et al., 2005 [73]	Mitoxantrone	0	Recurrent MG (12)			-	-	Procedure problem (25%), infection (8%)

Carpentier et al., 2006 [63]	CpG-ODN	I	Recurrent GBM (24)	1 mL/catheter	0.2 mL/h/catheter	-	-	Worsening of previous neurological condition (21%), partial seizures (21%), somnolence (8%), fever (21%), fatigue (25%), nausea (4%), lymphopenia (46%), ALT (25%), AST (4%)

Carpentier et al., 2010 [74]	CpG-ODN	II	Recurrent GBM (31)	1 mL/catheter 2 catheters	0.2 mL/h/catheter	3/31	28 wks	Worsening of previous neurological condition (65%), partial seizures (42%), general seizures (16%), fever (grade 2) (3%), fatigue (grade 2) (6%), hemorrhage leading to death 8 days after treatment (3%), lymphopenia (grade 2) (71%), lymphopenia (grade 3) (48%), ALT (10%)

Hau et al., 2007 [86]	TGF-*β*2 inhibitor Trabedersen	I/II	AA (5) GBM (19)	23-81 mL/cycle up to 10 cycles	0.24-0.32 mL/h	24	146.6 wks AA 44.0 wks GBM	Serious adverse events central and peripheral nervous system disorders (92%)

Bogdahn et al., 2011 [75]	TGF-*β*2 inhibitor Trabedersen	IIb	Recurrent/ refractory GBM, AA (145)	40 mL/cycle up to 11 cycles	0.24 mL/h	Not reported	AA: 39.1 mos (10 *μ*M) vs. 35.2 mos (80 *μ*M) vs. 21.7 mos (TMZ or PCV) GBM: 7.3 mos (10 *μ*M) vs. 10.9 mos (80 *μ*M) vs. 10 mos (TMZ or PCV)	Headache (10%), nervous system disorders (59-66%), depressed level of consciousness (4-12%), hemiparesis (22-27%), aphasia (10-15%), neurological symptom (8-17%), convulsion (8-12%), injury poisoning, and procedural complications (16-17%), infections and infestations brain abscess (7-12%), psychiatric disorders (6-12%), blood and lymphatic system disorders (5-8%)

Bruce et al., 2011 [78]	Topotecan	Ib	Recurrent GBM (10), AA (2), AE (2), AO (2)	40 mL	0.2 mL/h	-	-	Headache (31%), seizure (31%), worsened hemiparesis (31%), right-hand dyscoordination (13%), upper-extremity weakness (6%), poor wound healing (13%), intracerebral hemorrhage (6%), thrombocytopenia/leukopenia (13%), gastrointestinal symptoms (25%)

Desjardins et al., 2018 [87]	Recombinant poliovirus	II	Recurrent GBM (61)	3.25 mL	0.5 mL/h	-	12.5 mos (PVSRIPO) vs. 11.3 mos (Historical control)	Headache (52%), hemiparesis (50%), seizure (45%), dysphasia (28%), cognitive disturbance (25%), hemianopia (19%), confusion (18%), paresthesia (13%), fatigue (12%), nausea (10%)

Abbreviations. AA: anaplastic astroglioma; AE: anaplastic ependymoma; ALT: alanine aminotransferase; AST: aspartate aminotransferase; AO: anaplastic oligodendroglioma; GSC: gliosarcoma; MG: malignant glioma; n: number of patients; OA: oligoastrocytoma; pts: patients.
